# Attrition through Multiple Stages of Pre-Treatment and ART HIV Care in South Africa

**DOI:** 10.1371/journal.pone.0110252

**Published:** 2014-10-20

**Authors:** Matthew P. Fox, Kate Shearer, Mhairi Maskew, Gesine Meyer-Rath, Kate Clouse, Ian Sanne

**Affiliations:** 1 Center for Global Health & Development, Boston University, Boston, Massachusetts, United States of America; 2 Health Economics and Epidemiology Research Office, Department of Internal Medicine, School of Clinical Medicine, Faculty of Health Sciences, University of the Witwatersrand, Johannesburg, South Africa; 3 Department of Epidemiology, Boston University School of Public Health, Boston, Massachusetts, United States of America; 4 Vanderbilt Institute for Global Health, Vanderbilt University, Nashville, Tennessee, United States of America; 5 Clinical HIV Research Unit, Department of Internal Medicine, School of Clinical Medicine, Faculty of Health Sciences, University of the Witwatersrand, Johannesburg, South Africa; University of Washington, United States of America

## Abstract

**Introduction:**

While momentum for increasing treatment thresholds is growing, if patients cannot be retained in HIV care from the time of testing positive through long-term adherence to antiretroviral therapy (ART), such strategies may fall short of expected gains. While estimates of retention on ART exist, few cohorts have data on retention from testing positive through long-term ART care.

**Methods:**

We explored attrition (loss or death) at the Themba Lethu HIV clinic, Johannesburg, South Africa in 3 distinct cohorts enrolled at HIV testing, pre-ART initiation, and ART initiation.

**Results:**

Between March 2010 and August 2012 we enrolled 380 patients testing HIV+, 206 initiating pre-ART care, and 185 initiating ART. Of the 380 patients enrolled at testing HIV-positive, 38.7% (95%CI: 33.9–43.7%) returned for eligibility staging within ≤3 months of testing. Of the 206 enrolled at pre-ART care, 84.5% (95%CI: 79.0–88.9%) were ART eligible at their first CD4 count. Of those, 87.9% (95%CI: 82.4–92.2%) initiated ART within 6 months. Among patients not ART eligible at their first CD4 count, 50.0% (95%CI: 33.1–66.9%) repeated their CD4 count within one year of the first ineligible CD4. Among the 185 patients in the ART cohort, 22 transferred out and were excluded from further analysis. Of the remaining 163, 81.0% (95%CI: 74.4–86.5%) were retained in care through two years on treatment.

**Conclusions:**

Our findings from a well-resourced clinic demonstrate continual loss from all stages of HIV care and strategies to reduce attrition from all stages of care are urgently needed.

## Introduction

Since modeling demonstrated that strategies of massive HIV testing and treating patients immediately after being found HIV-positive could substantially impact HIV incidence [1]–[10], enthusiasm for increasing treatment thresholds has grown. More recently, demonstration of a 96% reduction in transmission risk from an infected partner with a CD4 count between 350–500 cells to an uninfected partner with immediate HIV treatment vs. waiting until a CD4≤250 (HPTN-052) has increased the motivation to increase treatment thresholds [11]. Because the risk of HIV transmission is substantially reduced with decreasing viral load [11]–[14], the hope is that increasing treatment thresholds could have an important impact on reducing HIV incidence. The World Health Organization now recommends that national programs provide first-line treatment to anyone with a CD4 cell count of less than 500 cells [15], revised from earlier thresholds of 350 and 200.

While it is hoped that increasing treatment thresholds will have a benefit for patients taking treatment in addition to a reduction in transmission, if patients cannot be retained in HIV care continuously from the time of testing positive through long term adherence to antiretroviral therapy (ART), such strategies may fail to achieve the expected benefits to the individual, let alone any population benefits. In addition as increasing thresholds means more people will be eligible for treatment, it may also divert resources that are critically needed for linking patients who test HIV positive to care and treatment sites and for retaining patients in both pre-ART and ART care. Retention in both pre-ART [16] and ART care [17], [18] have been shown to be sub-optimal.

Given the costs that are required for increasing treatment thresholds, it is important that we have a thorough understanding of retention in care under current guidelines so that we can monitor any differences associated with changing thresholds. While numerous estimates of retention on ART exist, few have data on retention from testing positive through pre-ART and on ART care within a single HIV care setting. We set out to estimate retention in three stages of HIV care within a single HIV care facility in Johannesburg, South Africa and to look for predictors of loss at each stage.

## Methods

### Study Site and Study Procedures

The study was conducted at the Themba Lethu HIV clinic in Johannesburg [19], [20]. Themba Lethu is a comprehensive public-sector HIV care and management site that also receives PEPFAR support through a South African NGO called Right to Care. Themba Lethu is located at the Helen Joseph Hospital and provides both HIV testing as well as pre-ART and ART care, as per the South African national HIV treatment guidelines. The site currently has >8,500 patients in pre-ART care, >11,000 patients on ART and conducts roughly 840 HIV tests per month. At the study’s start, guidelines called for treatment initiation at a CD4 count ≤200 or a WHO Stage IV condition [21]. In 2011 guidelines were updated to allow initiation with a CD4 count ≤350 [22].

At Themba Lethu, data on patients testing HIV positive at HIV counseling and testing (HCT) are kept in paper registers. Patients who test at the site are a mix of patients seeking self-initiated HCT as well as provider-initiated HCT from elsewhere in the hospital. Patients who test positive are sent from the HCT clinic to the HIV clinic for a blood draw for CD4 staging and asked to return in one week for the results. Those not eligible for ART begin in “wellness care” and have a paper file opened. Wellness patients are asked to return every six months for repeat CD4 testing, or more frequently when closer to the treatment threshold. Patients who are treatment eligible are asked to return for adherence and treatment readiness counseling over 2–3 weeks and then initiate treatment. Once a patient initiates ART, they are asked to return to the clinic every 1–3 months for the first twelve months for either drug pickups or medical visits.

Data on patients initiating ART care is kept in an electronic patient database. All pre-ART paper records are entered into the database at ART initiation. An effort is underway to enter all pre-ART records for all patients, but it is not complete. Accordingly, this study relied on both electronic and paper records to determine if patients returned for care.

To assess retention in all stages of HIV care we enrolled three distinct cohorts of patients at three different stages: a cohort of patients enrolled at the time of testing HIV-positive at HIV counseling and testing (referred to as the HCT cohort), a cohort of patients newly enrolled in pre-ART care after completing CD4 staging, regardless of ART eligibility, (referred to as the pre-ART cohort), and a cohort of patients newly initiating HIV treatment (referred to as the ART cohort). Patients who completed one stage of care were not followed after completing that stage of care. These cohorts roughly followed the published framework on pre-ART care [16] and additionally added a cohort post-ART initiation. Enrollment began on March 23, 2010 and was completed on August 19, 2012. We enrolled patients at care visits that were newly enrolled in pre-ART care (first CD4 count within 3 months of enrollment) and newly initiated on ART (on ART for no more than 1 month at the time of the interview). We attempted to recruit all patients eligible for care on the days where study staff were at the clinic, but as only one data collector was used who moved between part of the clinic (testing and treatment), enrollment was not done every day for each cohort. Due to sensitivity around receiving a positive HIV diagnosis, we enrolled HCT patients before they tested but limited our analysis to those who tested positive.

### Patient follow-up

Patients from all three cohorts went through an informed consent process and completed a questionnaire to look for predictors of attrition beyond what would be captured in the treatment database. Patients also provided identifying information to allow us to track their clinic records including a South African national ID number when possible. Upon completion of the questionnaire, participants followed the standard clinic schedule of procedures. In order to not influence patient retention, no active follow-up of patients was conducted by the research team. When resources allow, clinic staff attempt to phone patients who do not return for care.

Upon database closure, we attempted to match all enrolled eligible patients to the HIV care database using national ID (when provided, 82.7% of patients), name and date of birth. We then searched in paper records and registers for patients who could not be found in the dataset. Because all ART patients should have a clinic record, one ART patient with no records was excluded from analysis. For the HCT and pre-ART cohorts, not having a clinic record would identify the patient as lost to follow-up. We excluded patients who withdrew from the study (n = 2), and in the pre-ART cohort, who did not have a CD4 count (n = 34), who transferred out (n = 21) or whose first CD4 count was recorded outside +/−3 months from either the date of first visit or the interview date (n = 37).

### Analytic Methods

For the HCT cohort, loss to follow-up (LTF) was defined as not completing assessment for ART eligibility within 3 months of testing HIV positive. In the pre-ART cohort, LTF was defined as not initiating ART within 6 months of becoming eligible or, for those not eligible for ART, not repeating ART eligibility screening within 12 months of the previous ineligible staging. We note that the time frames used to allow patients to complete each stage of care are not necessarily ideal for patient care, but were chosen to balance the desires for fast, continual care but also to allow for the realities of patients’ lives [23]. Finally, for ART patients we use the clinic’s LTF definition of ≥3 months late for the next scheduled appointment. In the HCT cohort, the interview CD4 count was defined as the CD4 count closest to the interview date (84.3% were within +/−1 month).

We also present a secondary analysis using the time frames expected according to the clinic’s guidelines of completion of ART eligibility assessment within 1 week of testing positive, ART initiation within 1 month of the first eligible CD4 count, and repeat ART eligibility screening within 6 months of the previous ineligible staging. Follow-up time accrued from beginning of the stage until the earliest of loss to follow-up, death, transfer, completing 24 months of follow-up or dataset closure (September 30, 2013). To determine the vital status of patients lost, we searched their national ID number in the national vital registration database.

For each cohort (stage of HIV care) we present descriptive statistics using medians and interquartile ranges (IQRs) for continuous variables and proportions for categorical variables. Our primary analysis was to measure the proportion of patients who did not complete each stage of HIV care (either through death or loss). In order to examine predictors of not completing each stage, we used modified Poisson regression with robust error estimation to estimate the risk of not completing the stage with corresponding 95% confidence intervals. Age and sex were included in all of the models as was CD4 count in the pre-ART and ART model. Other possible confounders associated with not completing the stage (p<0.2) in univariate analyses were also adjusted for in the multivariate analyses.

### Ethical Clearance

Approval for analysis was granted by the Human Research Ethics Committee of the University of the Witwatersrand and by the Institutional Review Board of Boston University. Anonymized data can be requested from the corresponding author.

## Results

Enrollment of patients into all three cohorts was conducted from March 2010 through August 2012. We enrolled 380 patients testing HIV-positive at HCT, 206 patients initiating pre-ART care, and 185 patients initiating HIV treatment. We had 45 refusals in the HCT cohort, 37 refusals in the wellness cohort and 26 refusals in the ART initiation cohort.

### HIV Testing to Completing CD4 Staging

The first stage in the HIV care cascade requires patients to test for HIV. Those who test positive must have a blood draw for a CD4 count to determine ART eligibility and then return for the results. We enrolled 380 patients who tested HIV-positive. Of these, 56.1% were female, and the median age (IQR) was 35.8 (27.8–42.7) years (Table 1). A majority were unmarried (79.0%) and had some or completed high school (79.1%). Unemployment was common (37.7%) and over half (62.3%) required >30 minutes travel time to the clinic.

**Table 1:** Demographic and Clinical Characteristics of Three Cohorts of Patients Enrolled in Different Stages of HIV care in Johannesburg, South Africa.

	HCT Cohort			Pre-ART Cohort			ART Cohort		
Variable	Total	CompletedStage	Did NotComplete Stage	Total	CompletedStage	Did NotComplete Stage	Total	CompletedStage	Did NotComplete Stage
**Total N**	380 (100%)	147 (100%)	233 (100%)	206 (100%)	169 (100%)	37 (100%)	163 (100%)	132 (100%)	31 (100%)
**Sex**									
Missing	34	1	33	0	0	0	0	0	0
Male	152 (43.9%)	62 (42.5%)	90 (45.0%)	62 (30.1%)	51 (30.2%)	11 (29.7%)	33 (20.3%)	25 (18.9%)	8 (25.8%)
Female	194 (56.1%)	84 (57.5%)	110 (55.0%)	144 (69.9%)	118 (69.8%)	26 (70.3%)	130 (79.7%)	107 (81.1%)	23 (74.2%)
**Age** †									
Median (IQR) N	35.8 (27.8–42.7)	37.5 (32.1–43.8)	35.0 (26.1–42.1)	37.4 (31.7–43.5)	38.1 (31.7–43.8)	35.5 (31.8–39.6)	37.1 (31.5–43.9)	37.8 (31.8–43.4)	35.9 (28.5–46.0)
18–29.9	115 (30.3%)	31 (21.1%)	84 (36.1%)	40 (19.4%)	32 (18.9%)	8 (21.6%)	34 (20.9%)	26 (19.7%)	8 (25.8%)
30–34.9	57 (15.0%)	25 (17.0%)	32 (13.7%)	42 (20.4%)	33 (19.5%)	9 (24.3%)	29 (17.8%)	23 (17.4%)	6 (19.4%)
35–39.9	70 (18.4%)	31 (21.1%)	39 (16.7%)	42 (20.4%)	31 (18.3%)	11 (29.7%)	38 (23.3%)	31 (23.5%)	7 (22.6%)
40–44.9	67 (17.6%)	27 (18.4%)	40 (17.2%)	38 (18.5%)	35 (20.7%)	3 (8.1%)	25 (15.3%)	24 (18.2%)	1 (3.2%)
≥45	71 (18.7%)	33 (22.5%)	38 (16.3%)	44 (21.4%)	38 (22.5%)	6 (16.2%)	37 (22.7%)	28 (21.2%)	9 (29.0%)
**Country of birth**									
Missing	21	1	20	1	1	0	0	0	0
South Africa	339 (94.4%)	132 (90.4%)	207 (97.2%)	170 (82.9%)	137 (81.6%)	33 (89.2%)	140 (85.9%)	114 (86.4%)	26 (83.9%)
Other	20 (5.6%)	14 (9.6%)	6 (2.8%)	35 (17.1%)	31 (18.5%)	4 (10.8%)	23 (14.1%)	18 (13.6%)	5 (16.1%)
**Marital status**									
Missing	80	30	50	0	0	0	0	0	0
Unmarried‡	237 (79.0%)	102 (87.2%)	135 (73.8%)	168 (81.6%)	138 (81.7%)	30 (81.1%)	131 (80.4%)	104 (78.8%)	27 (87.1%)
Engaged or married	63 (21.0%)	15 (12.8%)	48 (26.2%)	38 (18.5%)	31 (18.3%)	7 (18.9%)	32 (19.6%)	28 (21.2%)	4 (12.9%)
**Education level**									
Missing	35	2	33	0	0	0	0	0	0
Primary school or less	72 (20.9%)	31 (21.4%)	41 (20.5%)	49 (23.8%)	40 (23.7%)	9 (24.3%)	34 (20.9%)	26 (19.7%)	8 (25.8%)
Some high school	130 (37.7%)	63 (43.5%)	67 (33.5%)	76 (36.9%)	61 (36.1%)	15 (40.5%)	80 (49.1%)	67 (50.8%)	13 (41.9%)
Completed high school or more	143 (41.5%)	51 (35.2%)	92 (46.0%)	81 (39.3%)	68 (40.2%)	13 (35.1%)	49 (30.1%)	39 (29.6%)	10 (32.3%)
**Employment status** ¥									
Missing	35	2	33	0	0	0	0	0	0
Employed	215 (62.3%)	103 (71.0%)	112 (56.0%)	132 (64.1%)	114 (67.5%)	18 (48.7%)	100 (61.4%)	85 (64.4%)	15 (48.4%)
Unemployed	130 (37.7%)	42 (29.0%)	88 (44.0%)	74 (35.9%)	55 (32.5%)	19 (51.4%)	63 (38.6%)	47 (35.6%)	16 (51.6%)
**Type of transport**									
Missing	80	30	50	0	0	0	0	0	0
Used a taxi	213 (71.0%)	97 (82.9%)	116 (63.4%)	186 (90.3%)	151 (89.4%)	35 (94.6%)	137 (84.0%)	111 (84.1%)	26 (83.9%)
Did not use a taxi	87 (29.0%)	20 (17.1%)	67 (36.6%)	20 (9.7%)	18 (10.6%)	2 (5.4%)	26 (16.0%)	21 (15.9%)	5 (16.1%)
**Travel time to clinic**									
Missing	80	30	50	0	0	0	2	1	1
≤30 minutes	113 (37.7%)	38 (32.5%)	75 (41.0%)	57 (27.7%)	48 (28.4%)	9 (24.3%)	34 (21.1%)	28 (21.4%)	6 (20.0%)
31–60 minutes	137 (45.7%)	61 (52.1%)	76 (41.5%)	86 (41.8%)	70 (41.4%)	16 (43.2%)	70 (43.5%)	58 (44.3%)	12 (40.0%)
>60 minutes	50 (16.7%)	18 (15.4%)	32 (17.5%)	63 (30.6%)	51 (30.2%)	12 (32.4%)	57 (35.4%)	45 (34.4%)	12 (40.0%)
**Household size**									
Missing	81	30	51	0	0	0	0	0	0
≤3 people	191 (63.9%)	71 (60.7%)	120 (65.9%)	127 (61.6%)	111 (65.7%)	16 (43.2%)	96 (58.9%)	74 (56.1%)	22 (71.0%)
≥4 people	108 (36.1%)	46 (39.3%)	62 (34.1%)	79 (38.4%)	58 (34.3%)	21 (56.8%)	67 (41.1%)	58 (43.9%)	9 (29.0%)
**CD4 count** 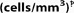									
Median (IQR) N	205 (99.5–344.5)	159 (75–245)	340 (160–505)	165 (85.5–256.5)	159 (85–247)	244.5 (151–446)	158 (95–198)	161.5 (106–199)	133 (91–178)
Missing	144	0	144	0	0	0	14	10	4
≤50	37 (15.7%)	27 (18.4%)	10 (11.2%)	29 (14.1%)	26 (15.4%)	3 (8.1%)	16 (10.7%)	14 (11.5%)	2 (7.4%)
51–100	22 (9.3%)	17 (11.6%)	5 (5.6%)	22 (10.7%)	20 (11.8%)	2 (5.4%)	25 (16.8%)	16 (13.1%)	9 (33.3%)
101–200	55 (23.3%)	46 (31.3%)	9 (10.1%)	67 (32.5%)	56 (33.1%)	11 (29.7%)	73 (49.0%)	63 (51.6%)	10 (37.0%)
>200	122 (51.7%)	57 (38.8%)	65 (73.0%)	88 (42.7%)	67 (39.6%)	21 (56.8%)	35 (23.5%)	29 (23.8%)	6 (22.2%)
**WHO Stage at ART Initiation**									
Stage I/II	–	–	–	–	–	–	98 (60.1%)	81 (61.4%)	17 (54.8%)
Stage III/IV	–	–	–	–	–	–	65 (39.9%)	51 (38.6%)	14 (45.2%)
**BMI at ART Initiation**									
Median (IQR)	–	–	–	–	–	–	22.8 (20.2–25.9)	22.6 (20.3–25.9)	22.9 (20.2–26.4)
Missing	–	–	–	–	–	–	10	8	2
<18.5	–	–	–	–	–	–	14 (9.2%)	11 (8.9%)	3 (10.3%)
18.5–24.9	–	–	–	–	–	–	94 (61.4%)	77 (62.1%)	17 (58.6%)
25–29.9	–	–	–	–	–	–	27 (17.7%)	23 (18.6%)	4 (13.8%)
≥30	–	–	–	–	–	–	18 (11.8%)	13 (10.5%)	5 (17.2%)
**Anemia at ART Initiation**									
Missing	–	–	–	–	–	–	10	10	0
None or mild							73 (47.7%)	58 (47.5%)	15 (48.4%)
Moderate or severe	–	–	–	–	–	–	80 (52.3%)	64 (52.5%)	16 (51.6%)

† Age at the time of the interview for the HCT cohort and the pre-ART cohort and at initiation for the ART cohort.

‡ Unmarried includes patients who reported being single, divorced/separated, and widowed.

¥ Employed includes patients who reported formal employment, informal employment, and self-employment. Unemployed includes those who reported no employment, studying, being retired, performing community service, and other.

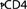
 at the time of the interview for the HCT cohort, first CD4 count conducted for the pre-ART cohort and at baseline for the ART cohort.

**Figure 1:**
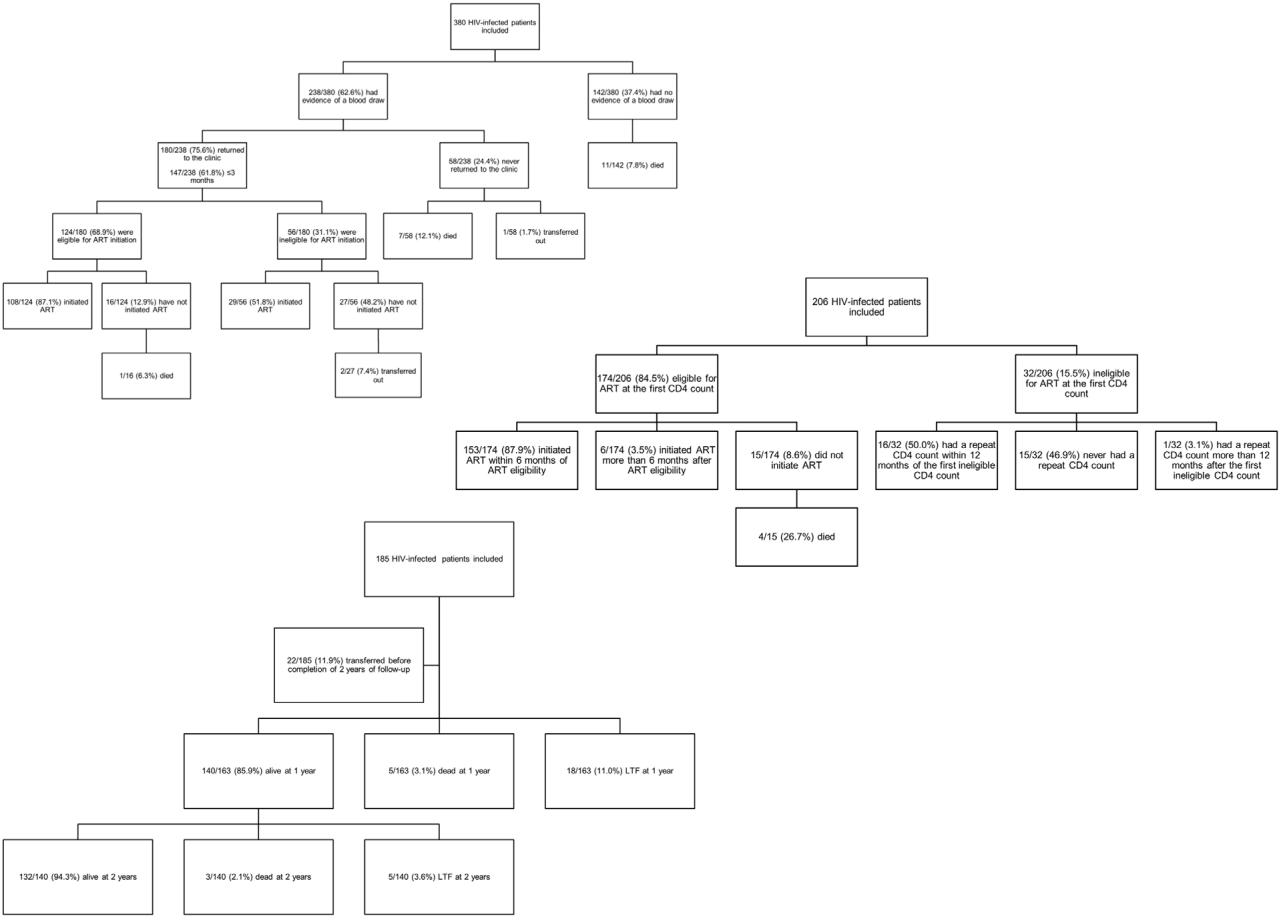
Flow Chart of Retention in Care of Three Cohorts of Patients Enrolled in Different Stages of HIV care in Johannesburg, South Africa.

Of the 380 patients enrolled at HIV-testing, we found no evidence of a blood draw for 142 patients and these were all considered failures. Of the remaining 238, 147 completed the stage for a total of 38.7% (95% CI: 33.9–43.7%) returning to complete eligibility staging within 3 months of testing, our definition of timely stage completion (Figure 1a). A total of 47.4% (an additional 33) ever returned after testing. Of those 200 (52.6%) who either never had a blood draw or never returned to the clinic, 9.0% (N = 18) were known to have died. We note, however, that because patients who test HIV positive could go elsewhere for CD4 staging and enrollment in care, this may overstate total attrition.

Among the 180 who returned to the clinic and had a CD4 count value recorded, a high percentage (68.9%, N = 124) were ART eligible. Treatment initiation was high amongst this group at 87.1% (95% CI: 80.3–92.2%). Of the 16 that did not initiate, 1 died before treatment initiation. While numbers were small, of the 56 patients who were not ART eligible, 51.8% (N = 29) became ART eligible during follow-up and started treatment.

Exploring predictors of failing to complete CD4 staging within 3 months, we found that less education was predictive of attrition (those completing some high school were 20% less likely to not complete the stage as those with primary school or less) (Table 2) as was employment status (unemployed were 29% more likely to not complete the stage as those employed). While there were few non-South Africans in our sample (N = 20), South Africans were more likely to not complete staging within 3 months as non-South Africans (aRR: 1.80; 95% CI: 0.87–3.72).

**Table 2:** Predictors of Attrition from Different Stages of HIV care in Johannesburg, South Africa.

	HCT Cohort			Pre-ART Cohort			ART Cohort		
Variable	Did not complete stage/N (%)	Unadjusted RR (95% CI)	Adjusted RR (95% CI)	Did not complete stage/N (%)	Unadjusted RR (95% CI)	Adjusted RR (95% CI)	Did not complete stage/N (%)	Unadjusted RR (95% CI)	Adjusted RR (95% CI)
**Sex**									
Male	90/152 (59.2%)	1.04 (0.87, 1.25)	1.10 (0.91, 1.33)	11/62 (17.7%)	0.98 (0.52, 1.86)	1.42 (0.73, 2.74)	8/33 (24.2%)	1.37 (0.68, 2.78)	1.30 (0.58, 2.88)
Female	110/194 (56.7%)	**Reference**	**Reference**	26/144 (18.1%)	**Reference**	**Reference**	23/130 (17.7%)	**Reference**	**Reference**
**Age**									
18–29.9	84/115 (73.0%)	1.36 (1.07, 1.74)	1.36 (1.00, 1.83)	8/40 (20.0%)	1.47 (0.56, 3.86)	1.43 (0.55, 3.74)	8/34 (23.5%)	0.97 (0.42, 2.22)	1.22 (0.47, 3.16)
30–34.9	32/57 (56.1%)	1.05 (0.77, 1.44)	1.17 (0.83, 1.66)	9/42 (21.4%)	1.57 (0.61, 4.03)	1.55 (0.60, 3.98)	6/29 (20.7%)	0.85 (0.34, 2.12)	0.94 (0.33, 2.64)
35–39.9	39/70 (55.7%)	1.04 (0.77, 1.41)	1.16 (0.82, 1.64)	11/42 (26.2%)	1.92 (0.78, 4.73)	1.98 (0.85, 4.65)	7/38 (18.4%)	0.76 (0.31, 1.82)	0.83 (0.31, 2.25)
40–44.9	40/67 (59.7%)	1.12 (0.83, 1.49)	1.23 (0.87, 1.75)	3/38 (7.9%)	0.58 (0.16, 2.16)	0.76 (0.19, 2.96)	1/25 (4.0%)	0.16 (0.02, 1.22)	0.23 (0.03, 1.71)
≥45	38/71 (53.5%)	**Reference**	**Reference**	6/44 (13.6%)	**Reference**	**Reference**	9/37 (24.3%)	**Reference**	**Reference**
**Country of birth**									
South Africa	207/339 (61.1%)	2.04 (1.04, 4.00)	1.80 (0.87, 3.72)	33/170 (1941%)	1.70 (0.64, 4.49)	–	26/140 (18.6%)	0.85 (0.37, 2.00)	–
Other	6/20 (30.0%)	**Reference**	**Reference**	4/35 (11.4%)	**Reference**	**–**	5/23 (21.7%)	**Reference**	**–**
**Marital status**									
Unmarried	135/237 (57.0%)	**Reference**	**Reference**	30/168 (17.9%)	**Reference**	**Reference**	27/131 (20.6%)	**Reference**	**–**
Engaged or married	48/63 (76.2%)	1.34 (1.12, 1.60)	1.40 (1.11, 1.75)	7/38 (18.4%)	1.03 (0.49, 2.17)	–	4/32 (12.5%)	0.61 (0.23, 1.61)	–
**Education level**									
Primary school or less	41/72 (56.9%)	**Reference**	**Reference**	9/49 (18.4%)	**Reference**	**–**	8/34 (23.5%)	**Reference**	**–**
Some high school	67/130 (51.5%)	0.91 (0.70, 1.18)	0.80 (0.60, 1.06)	15/76 (19.7%)	1.07 (0.51, 2.26)	–	13/80 (16.3%)	0.69 (0.32, 1.51)	–
Completed matric or more	92/143 (64.3%)	1.13 (0.89, 1.43)	0.91 (0.69, 1.21)	13/81 (16.1%)	0.87 (0.40, 1.89)	–	10/49 (20.4%)	0.87 (0.38, 1.97)	–
**Employment status**									
Employed	112/215 (67.7%)	**Reference**	**Reference**	18/132 (13.6%)	**Reference**	**Reference**	15/100 (15.0%)	**Reference**	**Reference**
Unemployed	88/130 (52.1%)	1.30 (1.09, 1.55)	1.29 (1.06, 1.58)	19/74 (25.7%)	1.88 (1.06, 3.36)	1.56 (0.87, 2.80)	16/63 (25.4%)	1.69 (0.90, 3.18)	1.40 (0.67, 2.94)
**Type of transport**									
Used a taxi	116/213 (54.5%)	**Reference**	**Reference**	35/186 (18.8%)	**Reference**	**–**	26/137 (19.0%)	**Reference**	**–**
Did not use a taxi	67/87 (77.0%)	1.41 (1.20, 1.67)	1.37 (1.11, 1.70)	2/20 (10.0%)	0.53 (0.14, 2.05)	–	5/26 (19.2%)	1.01 (0.43, 2.40)	–
**Travel time to clinic**									
≤30 minutes	75/113 (66.4%)	1.04 (0.81, 1.33)	0.88 (0.67, 1.15)	9/57 (15.8%)	0.83 (0.38, 1.82)	–	6/33 (17.7%)	0.84 (0.35, 2.03)	–
31–60 minutes	76/137 (55.5%)	0.87 (0.67, 1.12)	0.88 (0.68, 1.13)	16/86 (18.6%)	0.98 (0.50, 1.92)	–	12/70 (17.1%)	0.81 (0.40, 1.67)	–
>60 minutes	32/50 (64.0%)	**Reference**	**Reference**	12/63 (19.1%)	**Reference**	**–**	12/57 (21.1%)	**Reference**	**–**
**Household size**									
≤3 people	120/191 (62.8%)	1.09 (0.90, 1.33)	–	16/127 (12.6%)	0.47 (0.26, 0.85)	0.56 (0.30, 1.04)	22/96 (22.9%)	1.71 (0.84, 3.47)	1.52 (0.69, 3.36)
≥4 people	62/108 (57.4%)	**Reference**	–	21/79 (26.6%)	**Reference**	**Reference**	9/67 (13.4%)	**Reference**	**Reference**
**CD4 count (cells/mm^3^)**									
≤50	–	**–**	–	3/29 (10.3%)	**Reference**	**Reference**	2/16 (12.5%)	**Reference**	**Reference**
51–100	–	–	–	2/22 (9.1%)	0.88 (0.16, 4.82)	1.05 (0.19, 5.86)	9/25 (36.0%)	2.88 (0.71, 11.65)	2.01 (0.52, 7.79)
101–200	–	–	–	11/67 (16.4%)	1.59 (0.48, 5.27)	1.86 (0.58, 5.96)	10/73 (13.7%)	1.10 (0.27, 4.53)	1.00 (0.26, 3.85)
>200	–	–	–	21/88 (23.9%)	2.31 (0.74, 7.17)	2.43 (0.79, 7.49)	6/35 (17.1%)	1.37 (0.31, 6.07)	1.12 (0.25, 4.98)
**WHO Stage at ART Initiation**									
Stage I/II	–	–	–	–	–	–	17/98 (17.4%)	**Reference**	–
Stage III/IV	–	–	–	–	–	–	14/65 (21.5%)	1.24 (0.66, 2.34)	–
**BMI at ART Initiation**									
<18.5	–	–	–	–	–	–	3/14 (21.4%)	1.18 (0.40, 3.53)	–
18.5–24.9	–	–	–	–	–	–	17/94 (18.1%)	**Reference**	–
25–29.9	–	–	–	–	–	–	4/27 (14.8%)	0.82 (0.30, 2.23)	–
≥30	–	–	–	–	–	–	5/18 (27.8%)	1.54 (0.65, 3.63)	–
**Anemia at ART Initiation**									
None or mild	–	–	–	–	–	–	15/73 (20.6%)	**Reference**	–
Moderate or severe	–	–	–	–	–	–	16/80 (20.0%)	0.97 (0.52, 1.83)	–

### Initiation of Pre-ART Care Through Treatment Initiation

The next stage in pre-ART HIV care requires patients who completed CD4 staging and were ART eligible to start treatment and those not ART eligible to complete routinely scheduled CD4 eligibility staging visits. We enrolled 206 patients who had recently completed CD4 staging. Of those, 69.9% were female and the median age (IQR) was 37.4 (31.7–43.5) years (Table 1). As with the testing cohort, few subjects were married (18.5%) and about 40% completed high school. Unemployment was 35.9% in this cohort and over two-thirds (72.3%) required >30 minutes travel time to the clinic.

Of the 206 patients, 84.5% (95% CI: 79.0–88.9%) were ART eligible at their first CD4 count. Of those 174, 87.9% (95% CI: 82.4–92.2%) (N = 153/174) successfully completed the stage by initiating ART within 6 months while a further 6 (3.5%) initiated ART more than 6 months after learning of their eligibility. Of the 15 who never initiated, 4 (26.7%) are confirmed to have died. Among the 32 not ART eligible at their first CD4 count, 50.0% (95% CI: 33.1–66.9%) successfully remained in the stage by repeating their CD4 count within one year. Taken together 82.0% (N = 169/206) were adherent to the care protocol after initial CD4 staging completion.

For pre-ART patients, we found that a higher first CD4 count was predictive of failure to complete the stage (>200 vs. <50, aRR: 2.43; 95%CI: 0.79–7.49). We also found some evidence that males were more likely to fail to complete the stage than females (aRR: 1.42; 95% CI: 0.73–2.74), the only stage for which we identified even a modest gender association. Living in a smaller household was associated with completing the stage (aRR for ≤3 people vs. ≥4 people: 0.56; 95% CI: 0.30–1.04).

### Antiretroviral Therapy Initiation through 2 Years on ART

We enrolled 185 patients at treatment initiation. As enrollment occurred when the initiation CD4 threshold was <200, the median (IQR) CD4 count was 158 (95–198).

Of the 185, 22 patients transferred to another facility and are excluded from further analysis. After two years, among the remaining 163, 81.0% (95% CI: 74.4–86.5%)(N = 132) were retained through two years on treatment, while 23 were lost to follow-up and 8 died.

Patients without employment were somewhat more likely to not complete two years on treatment compared to those who reported employment in the formal and informal sectors or self-employment (aRR: 1.40; 95% CI: 0.67–2.94). We found very little else that was predictive of attrition from this stage, including age, sex, CD4 count and education, likely due to sample size.

### Results using alternative definitions

While the results presented above for the HCT and pre-ART cohorts pertain to definitions of loss described by Fox et al. [23], we repeated this analysis utilizing clinic guidelines for these two groups, which differ slightly. For patients testing positive in the HCT cohort, we defined LTF as failure to complete ART eligibility assessment within 1 week of testing positive while for the pre-ART cohort, we defined LTF as not initiating ART within 1 month of becoming eligible or, for those not eligible for ART, not repeating ART eligibility screening within 6 months of the previous ineligible staging.

Using these modified definitions, estimates of LTF increased considerably. Of the 380 patients enrolled in the HCT cohort, only 17 (4.5%; 95% CI: 2.7–6.9%) completed eligibility staging within 1 week of testing positive. Likewise, in the pre-ART cohort, just 25.9% (95% CI: 19.8–32.8%) of ART eligible patients initiated ART within 1 month of their eligible CD4 count and only 34.4% (95% CI: 19.6–51.9%) returned within 6 months to repeat eligibility screening.

## Discussion

Getting patients onto treatment earlier and maintaining them on ART lifelong has been an important goal of ART programs in an attempt to achieve benefits to the individual and their partners [24]–[27]. Our findings suggest that within our clinic population, 39% of patients who test positive complete CD4 staging within three months, 88% of patients who complete CD4 staging and are ART eligible initiate treatment within 6 months of eligibility and 81% of those starting treatment remain on treatment for two years. If we informally combine these three pieces of information by multiplying the probabilities, these estimates would suggest that approximately 28% of patients who test positive initiate treatment and remain on ART for at least the first two years. This does not account for attrition from those who are not treatment eligible at the time of entering pre-ART care, where monitoring is difficult and attrition is high. Thus, the overall picture of retention in this cohort is low in the pre-ART period, but better in the on-treatment era.

To put our estimates in context, we compared our results with the assumptions used in models of the potential benefits of test and treat strategies, approaches where massive testing is combined with treating all positives regardless of their CD4 count in order to reduce HIV incidence. In all these models, assumptions about retention on ART have varied. In the first analysis of universal test and treat, attrition was based on the Malawi national program, which saw an 8% immediate dropout rate and a 1–5% yearly dropout rate excluding deaths [1]. Another model estimated on ART attrition at 20% over the first year on treatment [4], in line with a previous review of on-ART retention [18]. A cost-effectiveness analysis of universal test and treat for discordant couples assumed a loss rate of 3.4/100 patient years, presumably during both pre-ART and ART periods [3]. While our attrition estimates include both death and loss to follow-up, these assumptions appear reasonably consistent with our findings and recent systematic reviews [18].

In terms of pre-ART linkage, one model used a linkage rate of 100% in order to test the maximum impact of universal test and treat and immediate ART initiation [2], while a second specified two-thirds of those testing positive completing linkage, a fair bit higher than our estimates [4]. A third model used a base-case scenario of 52% linkage, similar to that found in South Africa [28]. A fourth model assumed 41% loss between HIV testing and CD4 staging, and 54% loss between each 6-monthly pre-ART visit in their baseline scenario [29]. They found that increasing the proportion of patients receiving CD4 results was more cost-effective, while at expanded eligibility, improving ART retention was the most cost-effective option. Only universal test and treat with 80% testing and treatment initiation and zero ART dropout would result in cost savings, and only after 20 years [29]. A compilation of a series of models [2] tested the impact of varying assumptions about linkage to care and showed that the rate of linkage impacts overall expected benefits and cost. In analyzing the impact that interventions to improve uptake and retention could have compared to simply extending eligibility, they found improvements to the care cascade were more beneficial than increasing eligibility alone.

Our study is one of the few where multiple stages of retention are traced within a single treatment site. A similar analysis was conducted by Kranzer [30], who found roughly 60% linkage from testing to staging and two-thirds of those ART eligible were initiated. A second, conducted by Clouse that looked at both pre- and post-ART retention found roughly 70% of patients testing positive linked to CD4 staging and 74% of those ART eligible initiated [16] ^,^ [31]. Our findings on pre-ART attrition also are similar to what was found in two systematic reviews which found 59%–72% of patients testing positive linking to care and 62%–68% of ART eligible patients initiated treatment. Our findings are similar to these overall but with higher rates of initiation among those ART eligible and somewhat lower rates of linkage to pre-ART care of those testing HIV positive. These findings demonstrate that particularly in the pre-ART period, attrition is poor. Our finding of retention on ART at two years is somewhat higher, but in the range of findings from two systematic reviews of retention, which suggest two year retention at about 70% [17], [18], [32].

Taken together these findings suggest that test-and-treat programs must focus on retention, particularly in the pre-ART period in order to reduce morbidity, mortality and transmission. While we found good retention in the post-ART period, this study does not tell us whether in a test-and-treat strategy, pre-ART attrition will shift to the post-ART period or whether such strategies will reduce cascade-wide attrition. Future efforts to implement these strategies must pay close attention to this issue and measure progress over time.

Our study has several limitations that could impact the interpretation of our results. First, because we did not follow a large number of patients from testing through long-term treatment it is not clear whether it is appropriate to combine the three observed estimates of retention to summarize total retention. If patients who complete one stage differ from those we enrolled at the next stage, we may over or under estimate total attrition. Further, if patients leave one stage of care but return again at a later time point, we likely overestimate attrition, and as such our estimates should be considered estimates of timely stage completion. Second, as most subjects who entered pre-ART care were not eligible for ART, we cannot estimate retention in this stage of care. This would likely underestimate total attrition across pre-ART care. Third, our study was conducted at a treatment site with testing onsite. It is not clear whether the linkage rates we observed would be as high if the site was a standalone testing site where patients then had to link to a new site for care. Fourth, because our study relied on clinic record linkage to determine stage completion, patients who returned for care but did not have their records located would appear as patients who did not return when in fact they did. This would also likely overestimate attrition. Fifth, we excluded 34 pre-ART patients due to lack of a CD4 count as we could not assess whether or not they completed the stage under our definitions because we did not know if they were ART eligible. If these patients differed from the patients who did have a CD4 count, our results could be biased. Finally, South Africa does not use a national identification number for care. While we did collect this information, because not all clinics routinely use or collect this identifier, and because there are so many options for care in South Africa, we could not tell if patients who left care presented for care at another site. Not being able to account for these “silent transfers” means that our estimates of attrition may be overestimates. On the other hand, if patients who transferred sites are more likely to be lost from care than those who remain at a single site, overall estimates of attrition may be underestimates.

In conclusion, we found that linkage from testing to pre-ART care was 39%, retention in pre-ART care was 88% and two-year on ART retention was 81%. Much of the pre-ART attrition was among patients with high CD4 counts. These findings suggest substantial attrition in the pre-ART period, higher than that used in many models of test-and-treat. Future revision of these models should include sensitivity analyses to gauge the impact of such assumptions on overall predicted benefits.
